# Using Lignans from *Magnolia officinalis* Bark in the Assessment of the Quality of Dietary Supplements—The Application of ^1^H NMR and HPLC-DAD

**DOI:** 10.3390/ijms26041659

**Published:** 2025-02-15

**Authors:** Paweł Siudem, Aleksandra Wasiak, Agnieszka Zielińska, Violetta Kowalska, Katarzyna Paradowska

**Affiliations:** 1 Department of Organic and Physical Chemistry, Faculty of Pharmacy, Medical University of Warsaw, Banacha 1 Str., 02-097 Warsaw, Poland; oawasiak@gmail.com (A.W.); agnieszka.zielinska@wum.edu.pl (A.Z.); katarzyna.paradowska@wum.edu.pl (K.P.); 2 Department of Pharmaceutical Chemistry and Biomaterials, Faculty of Pharmacy, Medical University of Warsaw, Banacha 1 Str., 02-097 Warsaw, Poland; violetta.kowalska@wum.edu.pl

**Keywords:** magnolia officinalis bark, food supplements, ^1^H NMR, HPLC, PCA

## Abstract

In the present day, the use of dietary supplements is becoming increasingly common. This is due to two main reasons: a lack of nutrients from highly processed foods and the increasing popularity of dietary supplements. Supplements with anti-inflammatory properties, such as those containing magnolia bark extract, are particularly popular. Research has shown that magnolia bark extracts have anti-inflammatory, antioxidant, antidepressant, anti-anxiety, and anticancer effects, mainly due to the components magnolol and honokiol. With the increasing availability of magnolia bark extract dietary supplements, there is a need for fast analytical methods to assess their quality. This study aimed to compare the effectiveness of two analytical techniques, ^1^H NMR spectroscopy and HPLC-DAD chromatography, in evaluating dietary supplements containing magnolia bark extract. The results show that both techniques provide similar results and can be used for quality control. However, there is a significant difference between the actual and declared composition of the supplements, highlighting the importance of quality control for these products. To our knowledge, this is the first study showing the use of the ^1^H NMR method in the routine quantitative control of magnolia dietary supplements.

## 1. Introduction

In today’s rapidly changing world, the importance of ingredient supplementation is increasing. The daily diet is now recognised as one of the most important factors influencing the health of the human population. For this reason, there is a growing tendency for society to turn to natural methods described and proven by previous generations. One such science is Traditional Chinese Medicine, which has a history spanning centuries. It illustrates the beneficial effects of plants, including the magnolia *Magnolia officinalis*, syn. *Magnolia medicinalis*, a tree in the magnolia family (*Magnoliaceae*). This tree is found in the subtropics, in the eastern parts of North, Central and South America and South and West Asia [1].

The primary value of magnolias lies in their aesthetic appeal. They are estimated to be the oldest flowering plants on Earth, approximately 100 million years old. A distinction is made according to origin due to the multitude of magnolia species. Following this criterion, the Asian magnolias are distinguished as follows: Star magnolia (*M. stellata*), Japanese magnolia (*M. kobus*), Naked magnolia (*M. denudata*), Broad-leaved magnolia (*M. hypoleuca*, *M. obovata*), Willow magnolia (*M. salicifolia*), and Pink Glory magnolia (*M. Pink Glory*).

In Eastern medicine, different species have been recognised for centuries as plants with various properties. The 2010 Chinese Pharmacopoeia outlines three fundamental plant substances that are commonly used [2]. One of these is the substance of the medicinal magnolia, known as Houpo or Houpu [3]. In the case of magnolia, the following materials are employed: bark extracts, powdered bark, dried flower buds, and dried flowers [2].

These medicinal plant substances are attributed to a range of beneficial effects, including anti-inflammatory, anti-arrhythmic, antioxidant, antidepressant, anti-anxiety, anticancer, anaesthetic, and HSV inhibitory properties [4]. As expected, the most recent European Pharmacopoeia 11.0 contains monographs for the bark and flower of *M. medicinalis*. The most common method of ingestion is tea, which contains only magnolia bark. This type of infusion is traditionally used to relieve flatulence, diarrhoea, vomiting, food stasis, or asthmatic coughs.

In China, the magnolia flower is regarded as a symbol of purity and sincerity, while in Korea, it is accorded the status of a national symbol deployed for propaganda purposes. The magnolia bark is a source of a substantial number of potentially active compounds from a multitude of chemical groups, including alkaloids, coumarins, flavonoids, lignans, neolignans, phenylpropanoids, and terpenes [5,6].

In light of the most recent studies, it is clear that out of the approximately 200 compounds identified and isolated, two neolignans, honokiol and magnolol, deserve special attention [7,8].

Presently, a significant body of research is dedicated to investigating the effects of isolated compounds derived from the bark of *M. medicinalis*. Moreover, potential therapeutic applications have been expanded to encompass properties such as antimicrobial [9,10], analgesic [11], anti-inflammatory [12], antifungal [13], and anticancer effects [14,15]. Furthermore, there have been reports of isomers being employed in the treatment of conditions including depression, arrhythmia, asthma, gastrointestinal disorders, and even herpes simplex virus (HSV) infection [16,17,18].

The potential health benefits of *M. medicinalis* have led to the development of various products containing magnolia bark and its derivatives in the market. This study aimed to evaluate the quality and quantity of dietary supplements containing magnolia bark and its preparations, particularly those available in the Polish market. The active substances, honokiol and magnolol, were identified in all tested preparations using nuclear magnetic resonance spectroscopy (^1^H NMR) and high-performance liquid chromatography (HPLC). The percentage content of the two isomers in the magnolia bark was estimated using ^1^H qNMR and compared with data obtained from HPLC measurements. In studies published to date, the HLPC method is usually used to quantify magnolol and honokiol [19]. ^1^H NMR spectroscopy has been used for qualitative analysis of magnolia bark [20]. The goal of our project was to develop an ^1^H NMR method for the quantification of lignans in magnolia bark supplements and compare these results with a reference HPLC method.

## 2. Results and Discussion

### 2.1. ^1^H NMR Analysis

**Figure 1:**
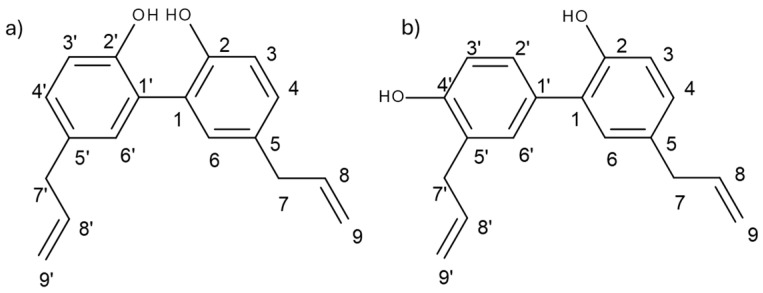
Structures of (**a**) magnolol and (**b**) honokiol with atom numbering.

To interpret the spectra of standard substances (magnolol and honokiol), ^1^H, ^13^C NMR spectra and 2D HSQC in CDCl_3_ were recorded. Figure 1 shows the structures along with the carbon numbering used in identification, and Table 1 and Table 2 summarise the chemical shift values (^1^H and ^13^C NMR spectra are presented in Supplementary Materials, Figures S1 and S2). Although ^1^H NMR spectra of magnolia lignans have been previously published [20,21], our objective was to rely on our results, supported by the interpretation of ^13^C and 2D spectra, as the definitive source of diagnostic signals.

To perform a qualitative and quantitative analysis of magnolol and honokiol based on ^1^H NMR spectra in the tested preparations, it was necessary to determine the diagnostic signals. A previously published study of magnolia bark of various origins also employed the ^1^H NMR method to examine extracts [20]. However, the precise quantitative outcomes derived from the NMR analysis were not disclosed, and the calibration curve method was not utilised. In contrast, our study required the selection of a diagnostic signal to generate a calibration curve. The comparison of the ^1^H NMR spectra recorded for pure isomers present in the bark of *M. officinalis* shows that many signals for honokiol and magnolol have similar chemical shift values (as indicated by the structural formulas of the compounds in Figure 1). In a mixture of these compounds, it is difficult to assign a given signal to a specific isomer. Compounds can only be distinguished based on the chemical shift value for protons from the CH_2_ group.

**Figure 2:**
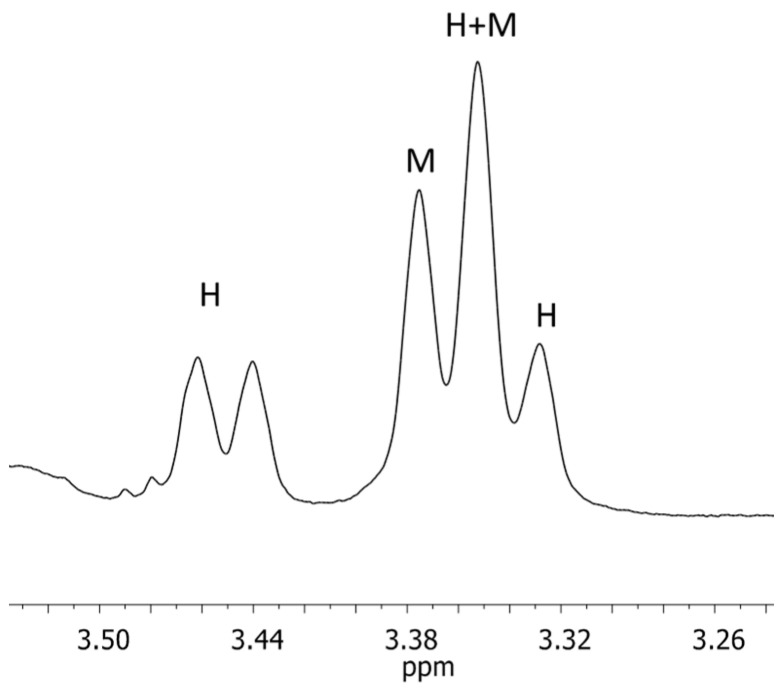
A fragment of the ^1^H NMR spectrum of honokiol and magnolol with diagnostic signals.

A doublet (δH 3.37 ppm, J = 6.7 Hz) was selected as diagnostic marker for magnolol (M). The signal arises from four equivalent protons marked as 7 and 7′. In the case of honokiol (H), signals from the aliphatic range (approx. 3.5 ppm) were also selected as a diagnostic signal. It is a doublet of doublets, in which each component corresponds to one group of two equivalent protons. The first (δH 3.46 ppm, J = 6.36 Hz) comes from protons 7′, while the second (δH 3.55 ppm, J = 6.68 Hz) corresponds to protons 7. The signals selected for both isomers partially overlap, creating a characteristic triplet consisting of a doublet from magnolol (δH = 3.37 ppm) and a doublet from honokiol (δH = 3.35 ppm), shown in Figure 2.

For quantitative and qualitative analysis, the evaporated extracts were dissolved in a mixture of chloroform and deuterated chloroform (1:1 *v*:*v*). This configuration ensured sufficient dissolution of the tested compounds in the smallest possible volume of the solution.

**Figure 3:**
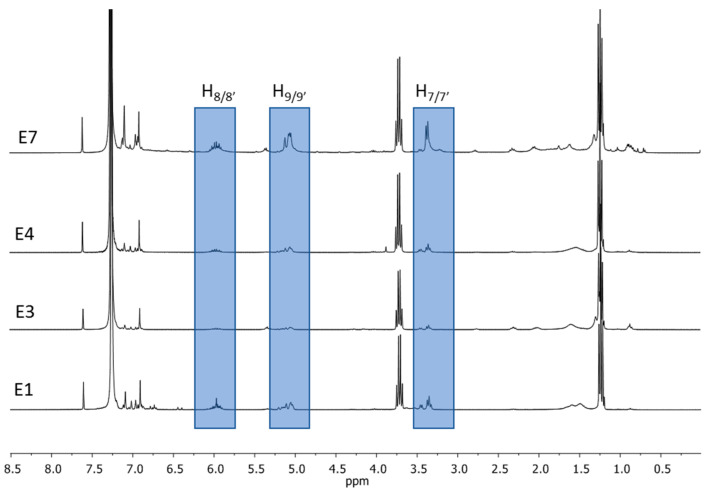
^1^H NMR spectra in CDCl_3_ registered for **E1**, **E3**, **E4**, and **E7**.

The recorded ^1^H NMR spectra of the solution for all preparations were compared with the spectra of standard substances: magnolol and honokiol (food supplement samples are labelled as **E1**–**E7**; more information is included in the table in Section 3). The **E1**, **E3**, **E4**, and **E7** ^1^H NMR spectra (of preparations containing powdered magnolia bark, Figure 3), apart from the signal characteristic of the determined isomers and of the solvents, show a number of signals in the aliphatic range. They come from the -CH_2_- groups of long-chain fatty acid molecules with 16 or 18 carbon atoms [20]. Polymers of these compounds create a specialised plant tissue—cutin—which performs protective functions in plants [22].

**Figure 4:**
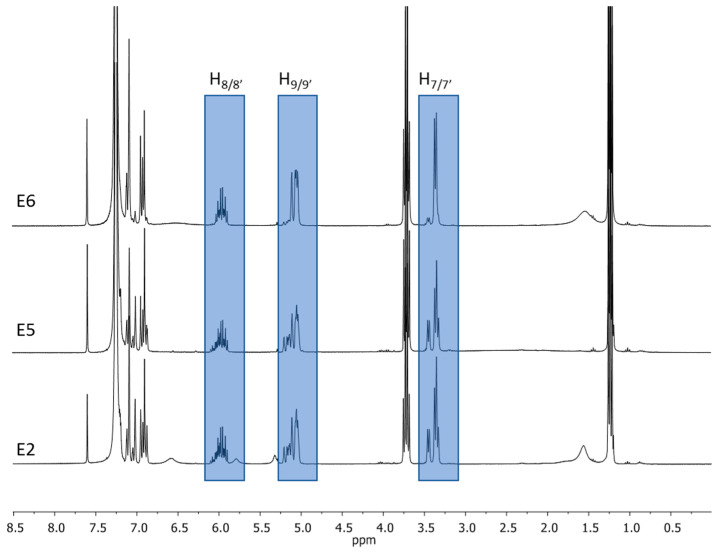
^1^H NMR spectra in CDCl_3_ registered for **E2**, **E5**, and **E6**.

Dietary supplements containing mainly magnolol and honokiol were devoid of high-molecular substances originating from the bark, which is reflected in the lack of signals arising from fatty acids in proton NMR spectra (Figure 4). This fact made it possible to divide the preparations into two groups:

- Those containing powdered magnolia bark (or bark’s extract with low honokiol/magnolol quantity), which includes **E1**, **E3**, **E4**, and **E7** (Figure 3);
- Those containing purified extracts with a high content of isomers: magnolol and honokiol, including **E2**, **E5**, and **E6** (Figure 4).

A preliminary analysis of the ^1^H NMR spectrum can provide valuable insights into the quality of the raw material, even without the need for lignan quantification.

The next stage was to confirm the presence of both isomers, magnolol and honokiol, in the tested supplements. The signals shown and described in Figure 2 were assigned in the spectra recorded for the samples. The presence of both isomers in each sample is confirmed, and the difference in intensity indicates the different ratios of the content of magnolol and honokiol in the supplements. Moreover, this result suggests the need to quantify the isomers.

**Figure 5:**
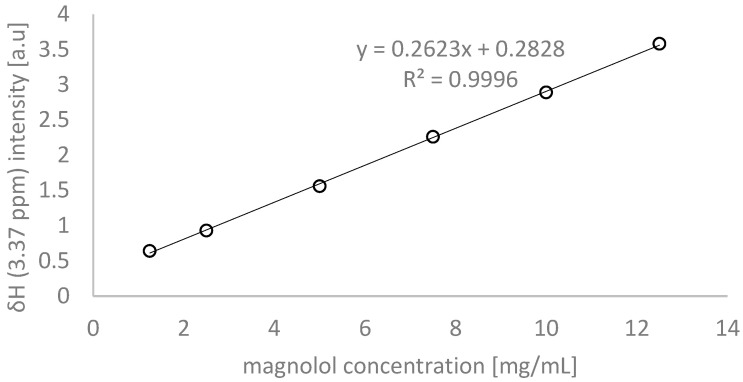
The calibration curve for ^1^H NMR quantification of magnolol.

The percentage of magnolol content was determined based on the selected signals and the prepared calibration curve (Figure 5). This was done to determine the exact quantity of the capsules, ground bark, and micronised tablet. The results are presented in Table 3. The highest magnolol content was found in **E5**, a powdered extract. The lowest amount of magnolol was determined in **E3**, the capsule containing the powdered bark (Table 3). Then, based on the standard curve drawn for magnolol, the sum of magnolol and honokiol was calculated using a signal intensity of 3.35 ppm. Since honokiol and magnolol have equal molar masses, and the signal at 3.35 ppm comes from the same number of protons of honokiol and magnolol, it was possible to use a single standard curve to determine the sum of these compounds. The results are shown in Table 3.

Furthermore, the lignan content as determined by ^1^H NMR is consistent with the results obtained from the HPLC measurements (see Section 2.3). Consequently, the NMR method can also be utilised in routine control, including quantitative control of dietary supplements from *M. officinalis* bark.

### 2.2. PCA

**Figure 6:**
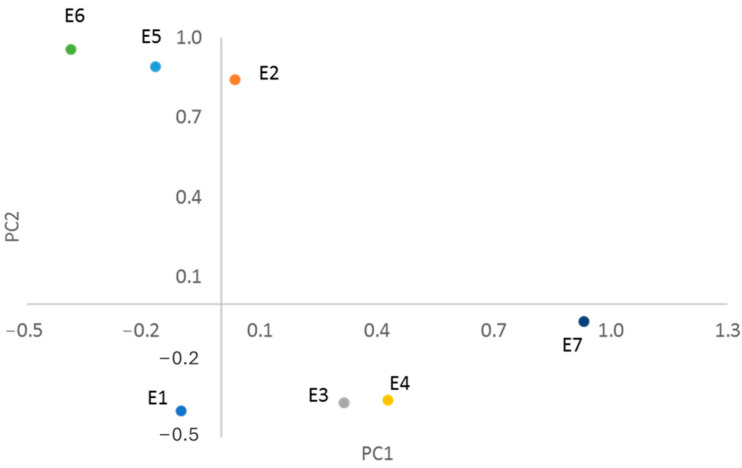
Score plot of PC1 vs. PC2 for studied supplements based on ^1^H NMR spectra processing.

The next stage of the process involved the principal component analysis (PCA) based on the ^1^H NMR spectra. Subsequently, each spectrum was divided into ranges following normalisation, and the intensities within each range were employed as the input for the chemometric analysis. The analysis demonstrated that the initial two variables, PC1 and PC2, collectively explained 63% of the total variance (40% and 23%, respectively). Consequently, only these were employed for interpretation. Figure 6 illustrates the clustering of points corresponding to the studied supplements (**E1**–**E5**) in the new coordinate system reduced to the two variables PC1 and PC2.

**Figure 7:**
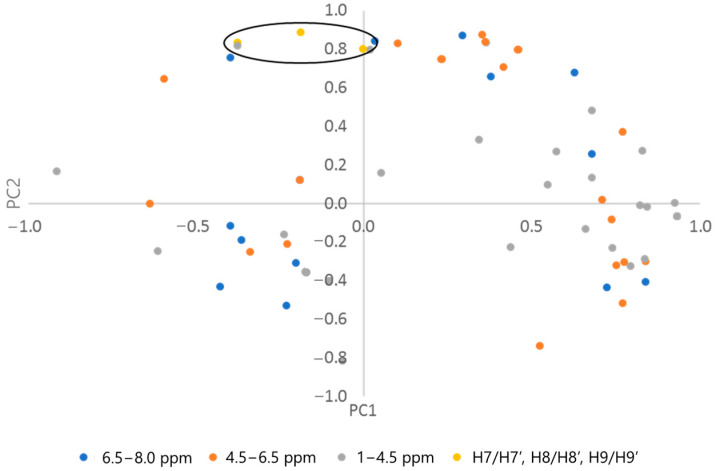
The loadings plot of the PCA grouped into chemical shift ranges. For readability, the points corresponding to the chemical shift of H7/H7′, H8/H8′, and H9/H9′ protons are listed (circular yellow points).

The graph illustrates that the points corresponding to supplements **E2**, **E5,** and **E6** are situated above the Y-axis, at the upper end of the graph, while those corresponding to extracts **E1**, **E3**, **E4,** and **E7** are located at the lower end (below the X-axis). This differentiation is concurrent with the quantification of magnolol content (see Table 4). The extracts obtained from supplements **E2**, **E5,** and **E6** were rich in magnolol, with concentrations above 30%. In contrast, **E1**, **E3**, **E4,** and **E7** contained relatively low levels of magnolol, with concentrations no greater than 2%. Therefore, the newly introduced variable, PC2, is defined as the quantitative content of magnolol in the extracts. This is evident from an examination of Figure 7, which illustrates the distribution of loadings in the PCA coordinate system. The points corresponding to the chemical shift values H7/H7′, H8/H8′, and H9/H9′ have the most significant influence on the positioning of the extracts studied, situated in the upper part of the graph. These are indicated on the graph with an ellipse.

It is evident that the positioning of the points on the X plot in relation to the Y-axis is correlated with the intensity of the signals observed in the 1–4.5 ppm spectral range. The unpurified magnolia bark (**E7**), which contains waxes and fatty compounds, exhibits a multitude of signals within this spectral range. In a study of extracts from disparate *Strychnos* species, the same correlation was identified for the extracts exhibiting the highest concentration of fatty compounds (obtained from seeds) [23]. Extracts purified to varying degrees of these compounds from the bark exhibited lower signal intensities. The new variable PC1 thus represents the quality of the extract, expressed as the degree of purification from the fatty compounds present in the bark. All supplements in capsules, tablets, or powder forms were characterised by a higher degree of purification from fatty derivatives.

In conclusion, it can be stated that ^1^H NMR analysis is a suitable method for the routine and rapid quality analysis of *Magnolia officinalis* bark supplements. Furthermore, the determination of signal intensities corresponding to the signals H7/H7′, H8/H8′, and H9/H9′ and the aromatic region 1–4.5 ppm is sufficient to determine the quality of the composition in a routine inspection.

### 2.3. HPLC-DAD

**Figure 8:**
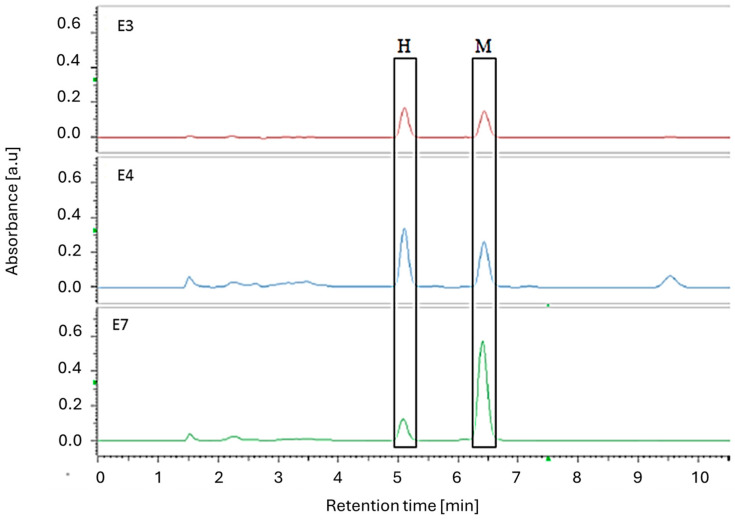
HPLC chromatograms (290 nm) of magnolia bark supplement extracts **E3**, **E4,** and **E7**.

The HPLC-DAD analysis was performed on all of the tested samples (chromatograms are presented in Supplementary Materials, Figure S3). Both isomers, honokiol and magnolol, were found in the dietary supplements and the prepared magnolia bark extract. This was confirmed by the presence of two peaks with retention times corresponding to these compounds (see Figure 8). Additionally, in the chromatograms for preparations **E3** and **E4**, as well as for the ethanolic extract of magnolia bark (**E7**), there are low-intensity peaks at the beginning of the chromatogram, indicating a low degree of processing of the plant substance.

**Figure 9:**
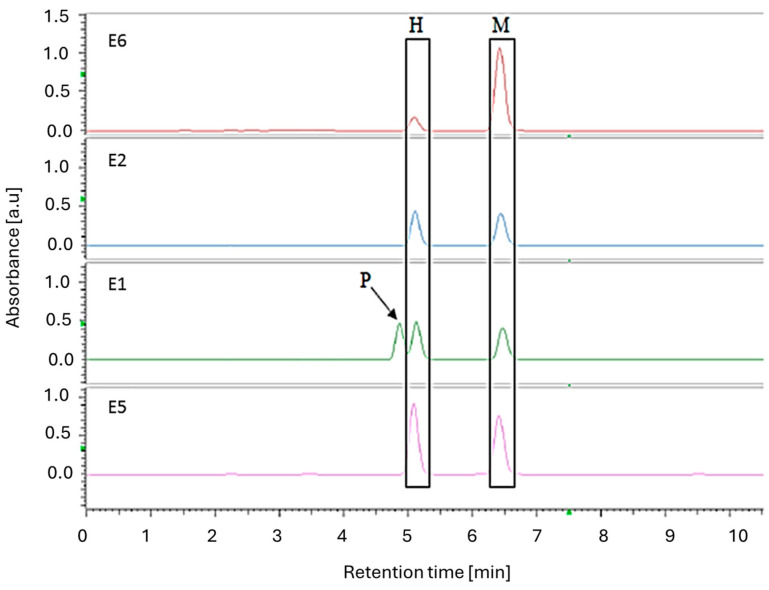
HPLC chromatograms (290 nm) of magnolia bark supplement extracts **E1**, **E2**, **E5,** and **E6**; the letter P indicates the peak corresponding to piperine, an additive to supplement **E1**.

The chromatograms (Figure 9) recorded for samples **E2**, **E5,** and **E6** exhibit only two peaks, whose retention times align with those of the standards. In the case of formulation **E1**, in addition to the two peaks characteristic of the lignans, a third peak is present with a retention time of RT = 4.85 min. This third peak is consistent with the manufacturer’s declaration and indicates that it is derived from piperine (it was confirmed by analysis of UV spectrum), a component in this dietary supplement commonly used as a bioenhancer [24].

The chromatograms confirmed the presence of both isomers in each sample, with varying content and ratios of magnolol and honokiol in the supplements. The results of HPLC quantification are summarised in Table 4.

Furthermore, the number of peaks and the corresponding retention times allowed us to divide the preparations into three groups:

1. Those containing powdered magnolia bark in the formulation, which included the prepared extract (**E3**, **E4**, **E7**);
2. Those containing purified extracts with a high content of magnolol and honokiol isomers (**E2**, **E5**, **E6**);
3. Those containing purified extracts with a high content of the isomers magnolol and honokiol with the addition of piperine—**E1**.

This corresponds to the classification made based on the results of ^1^H NMR studies.

The manufacturers’ failure to declare the qualitative and quantitative isomer content, as required, proved problematic. Moreover, the study used powdered magnolia bark, which, according to the standardisation outlined in the European Pharmacopoeia, is required to contain a minimum of 2.0% of combined honokiol and magnolol. Because the material used in this study was varied, it was necessary to summarise the content of each isomer and compare the data with the declared values (the table in the Section 3)).

The results demonstrate that the highest concentration of the combined isomers was observed in the powdered product designated as **E5**, while the lowest concentration was observed in the capsules **E2**. After accounting for standard deviations, only one preparation exhibited a concentration aligned with the declared amount (**E5**). The preparations with the lowest concentrations of the isomers were **E1**, **E3**, and **E4**, which do not specify the content of magnolol and honokiol. These preparations contained the isomers at concentrations below 3%.

## 3. Materials and Methods

The research material included dietary supplements containing magnolia bark or its extracts and magnolia bark itself. Dietary supplements and bark available on the Polish market were purchased in a herbal store, online, or obtained from the manufacturer. Summary information about the tested samples is presented in Table 5. The following substances were also used in the study: magnolol (ID: Y0001289) and honokiol (ID: Y0001290). These were purchased from Sigma-Aldrich, St. Louis, MO, USA.

### 3.1. Sample Preparation

The samples were prepared by re-extraction. The appropriate amount of the supplement, determined based on solubility analysis, was weighed into a conical flask. Extraction was performed with 99.8% ethanol and shaken on a rotary shaker at 250 rpm for 45 min. Then, the obtained extracts were filtered into round-bottomed flasks. The filters and conical flasks were washed with 5 mL of ethanol. The clear extract obtained was evaporated on a vacuum evaporator.

For HPLC analysis, the obtained extracts were dissolved in ethanol and diluted if necessary. For the qualitative and quantitative determination of magnolol and honokiol using NMR spectroscopy, the samples were dissolved in 1 mL of chloroform (CDCl_3_) stabilised with 2-methylbut-2-ene. A total of 0.5 mL of the obtained solutions was mixed with 0.5 mL of deuterated chloroform. The mixture prepared in this way was transferred to an NMR tube, and the ^1^H NMR spectrum was recorded.

### 3.2. ^1^H NMR Analysis

All spectra were recorded in solution (CDCl_3_) on a Varian VNMRS 300 MHz Oxford spectrometer using the standard Varian pulse program, with a relaxation delay of 1 s, an acquisition time of 2 s, and a number of scans of 64. The spectra were prepared using the MestReNova 11 program (Santiago de Compostela, Spain). Quantitative determination using nuclear magnetic resonance spectroscopy was performed using the calibration curve method.

To prepare the standard curve (details in Table 6), a series of solutions containing magnolol were prepared with the following concentrations: 10 mg/mL, 5 mg/mL, 2.5 mg/mL, and 1.25 mg/mL. ^1^H NMR spectra were recorded for all prepared samples, analysing the doublet intensity (δH = 3.37 ppm) and plotting a standard curve.

### 3.3. HPLC Analysis

For quantitative measurements of magnolol and honokiol, a Hitachi Chromaster HPLC system with a DAD diode detector (model 5430), a gradient pump (model 5160), and a column thermostat (model 5310) was used. The ethanol solutions were filtered into vials and placed in a thermostatically controlled autosampler. Samples were injected in volumes of 20 μL onto a Merck Purospher STAR RP-18e column (250 mm × 4.6 mm, 5 μm). Chromatographic separations were carried out at 30 °C, under isocratic conditions, with a mobile phase of acetonitrile/0.2% formic acid 75/25 (*v*/*v*), a flow rate of 1 mL/min, and a run time of 15 min. Each compound was detected at a wavelength of λ = 290 nm. The obtained values of retention times and areas under the peaks were compared with standard curves for magnolol and honokiol (details are summarised in Table 7). All measurements were made in triplicate.

The limit of detection (LOD) and limit of quantification (LOQ) were determined using the following formulas: LOD = 3.3 × σ/S and LOQ = 10 × σ/S (σ—the standard deviation of the y-intercept, S—the slope of the calibration curve). The % RSD (relative standard deviation) for intraday variation remained below 2%.

## 4. Conclusions

The combination of proton nuclear magnetic resonance (^1^H NMR) spectroscopy, which provides a comprehensive metabolite profile, and high-performance liquid chromatography (HPLC), targeting two specific biomarkers, represents an effective approach for quality control of *Magnolia officinalis* cortex and, consequently, of supplements containing this plant extract. Additionally, the PCA identified signals that can be used in the routine quality analysis of dietary supplements containing *M. officinalis* bark extract. This is particularly important in the context of studies examining the efficacy of supplements containing magnolia bark extract in chemoprevention [25], as the composition of magnolia bark supplements has not yet been widely studied. So far, the HPLC method has been routinely used to determine lignans in magnolia bark. Our results show that using the ^1^H NMR method gives results that are consistent with chromatographic measurements, and also provides additional information to differentiate the sample, such as the residual fatty substances.

## Figures and Tables

**Table 1:** ^1^H and ^13^C chemical shift values of magnolol.

Atom No.	δH [ppm] (Mult, J)	δC [ppm]
1	-	126.3
2	-	150.8
3	6.92 (1H. d. J = 1.48 Hz)	116.6
4	7.06 (1H. dd. J = 10.11. 1.72 Hz)	129.3
5	-	127.7
6	7.21 (1H. s)	130.2
7	3.35 (2H. d. J = 6.68 Hz)	39.4
8	6.01 (1H. m)	136.0
9	5.15 (4H. m)	115.6
1′	-	128.8
2′	7.24 (1H. d. J = 2.11 Hz)	128.6
3′	6.90 (1H. d. J = 6.55 Hz)	117.0
4′	-	154.0
5′	-	132.2
6′	7.02 (1H. d. J = 1.94 Hz	131.2
7′	3.46 (2H. d. J = 6.36 Hz)	35.2
8′	6.01 (1H. m)	137.8
9′	5.15 (4H. m)	115.6

**Table 2:** ^1^H and ^13^C chemical shift values of honokiol.

Atom No.	δH [ppm] (Mult, J)	δC [ppm]
1, 1′	-	123.4
2, 2′	-	151.2
3, 3′	6.97 (2H. bs/d. J = 8.25 Hz)	116.61
4, 4′	7.08 (2H. d. J = 2.00)	130.1
5, 5′	-	133.2
6, 6′	7.15 (2H. dd. J = 10.44; 2.19 Hz)	131.1
7, 7′	3.37 (4H. d. J = 6.7 Hz)	39.3
8, 8′	5.97 (2H. m)	137.5
9, 9′	5.09 (4H. m)	115.8

**Table 3:** The percentage content of magnolol in the tested supplements determined by using the ^1^H NMR method.

No.	Magnolol Content [%] by ^1^H NMR ± SD	Sum of Magnolol and Honokiol [%] by ^1^H NMR ± SD
**E1**	1.0 ± 0.02	3.23 ± 0.03
**E2**	31.8 ± 0.08	58.31 ± 0.10
**E3**	0.1 ± 0.00	0.27 ± 0.03
**E4**	0.3 ± 0.01	0.60 ± 0.10
**E5**	49.1 ± 0.10	92.10 ± 0.15
**E6**	44.0 ± 0.08	67.07 ± 0.11
**E7**	2.0 ± 0.04	2.13 ± 0.06

**Table 4:** The percentage content of honokiol and magnolol in the tested supplements determined by using the HPLC method.

No.	Honokiol Content [%]	Magnolol Content [%]	Sum of Magnolol and Honokiol [%]
**E1**	1.55 ± 0.19	1.35 ± 0.06	2.90 ± 0.16
**E2**	28.19 ± 2.10	27.48 ± 1.82	55.67 ± 3.92
**E3**	0.13 ± 0.01	0.12 ± 0.01	0.25 ± 0.02
**E4**	0.47 ± 0.00	0.40 ± 0.01	0.87 ± 0.01
**E5**	49.14 ± 1.27	43.05 ± 1.20	92.19 ± 2.48
**E6**	9.86 ± 0.18	63.95 ± 0.64	73.81 ± 0.47
**E7**	0.39 ± 0.01	1.80 ± 0.07	2.19 ± 0.07

**Table 5:** Information on studied products provided by manufacturers.

No.	Form	Declared Content
**E1**	Tablets	magnolia bark extract 6% 350 mg (honokiol 21 mg), black pepper extract 95% 5 mg
**E2**	Capsules	225 mg of *M. officinalis* bark extract (202.5 mg honokiol)—up to 90% of honokiol
**E3**	Capsules	magnolia bark (*M. officinalis*) 400 mg
**E4**	Capsules	*M. officinalis* extract standardised to 90% honokiol and magnolol 300 mg
**E5**	Powder	40% honokiol 50% magnolol
**E6**	Powder	extract containing 80% magnolol
**E7**	Bark	the minimum assumed by the European Pharmacopoeia 11 was adopted, i.e., 2.0%.

**Table 6:** Validation parameters of ^1^H NMR method for quantification.

Compound	δH of Signal [ppm]	Calibration Curve	R2	Linear Range (mg/mL)	LOD (mg/mL)	LOQ (mg/mL)
Magnolol	3.3.7	I = 0.2623c + 0.2828	1.000	1.25–10	0.26	0.78

**Table 7:** Validation parameters of HPLC method for quantification.

Compound	RT (min)	Calibration Curve	R2	Linear Range (mg/mL)	LOD (mg/mL)	LOQ (mg/mL)
Honokiol	5.2 ± 0.2	A = 17,065,311c + 16,355	1.000	0.025–0.40	0.011	0.035
Magnolol	6.8 ± 0.2	A = 16,112,707c + 20,558	0.999	0.025–0.40	0.004	0.012

## Data Availability

The original contributions presented in this study are included in the article/Supplementary Material. Further inquiries can be directed to the corresponding author.

## Supplementary Materials

The supporting information can be downloaded at https://www.mdpi.com/article/10.3390/ijms26041659/s1.
