# Supplementary material for: Using Lignans from Magnolia officinalis Bark in the Assessment of the Quality of Dietary Supplements—The Application of 1H NMR and HPLC-DAD
Source: Int J Mol Sci. 2025 Feb 15;26(4):1659. doi: 10.3390/ijms26041659 (PMC11855702; doi:10.3390/ijms26041659)
Supplement: Supplementary file 1 [file ijms-26-01659-s001.zip › ijms-3463481-supplementary.pdf]

Lignans from *Magnolia officinalis* bark in the assessment of the quality of dietary supplements – application of ^{1}H NMR and HPLC-DAD

Paweł Siudem, Aleksandra Wasiak, Agnieszka Zielińska, Violetta Kowalska, Katarzyna Paradowska

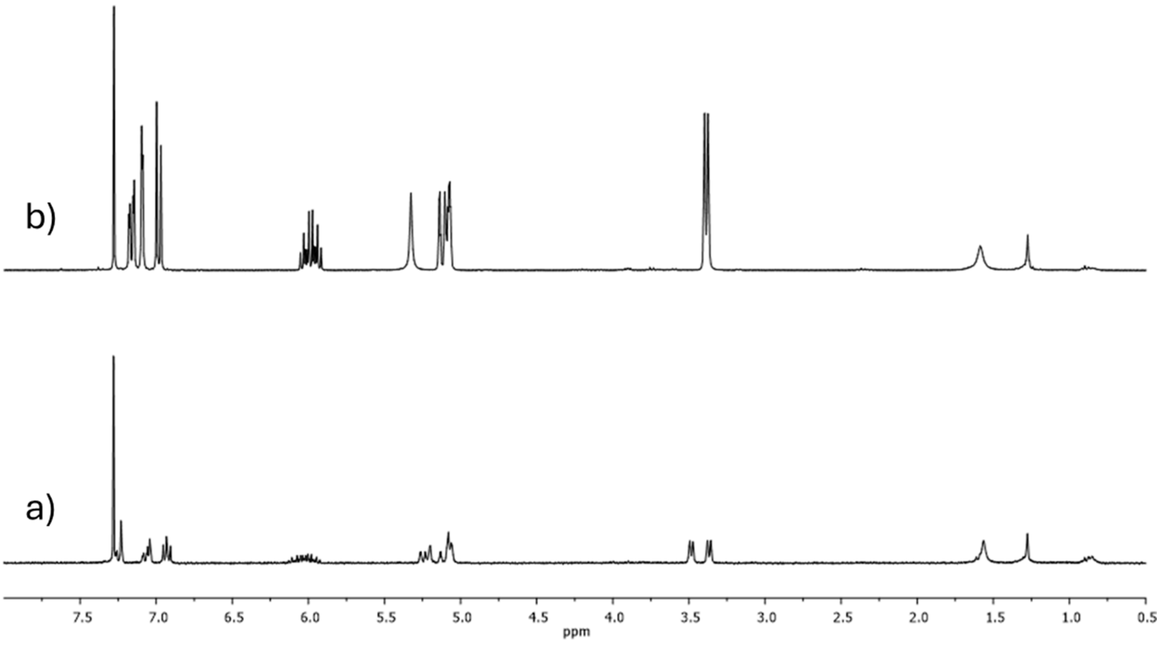

Figure S1. ^{1}H NMR spectra of a) magnolol and b) honokiol in $CDCl_3$.

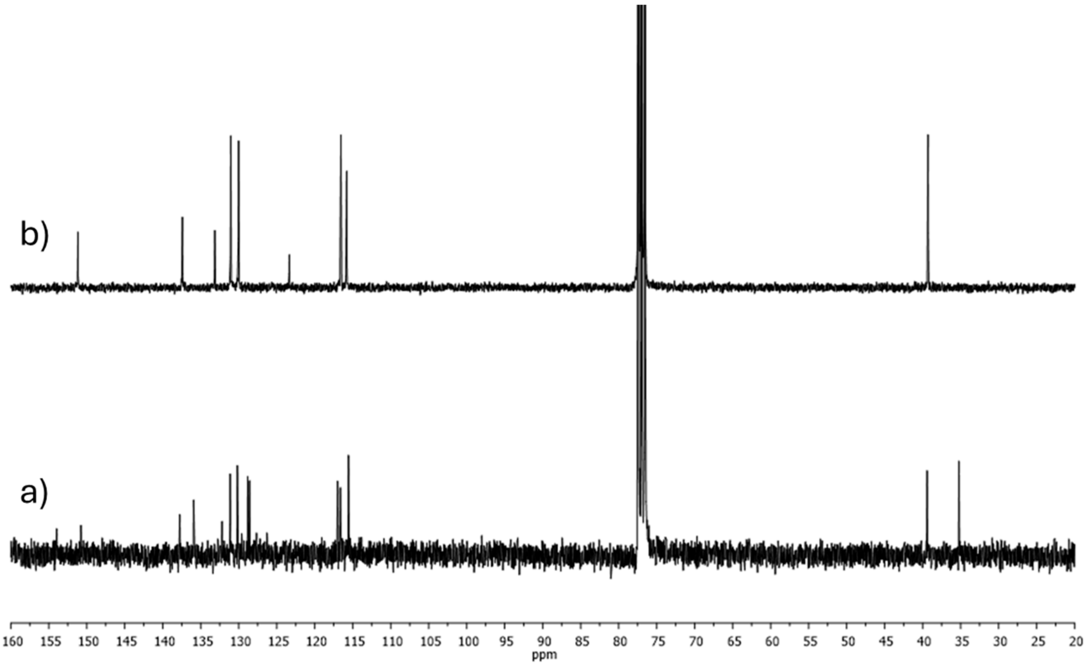

Figure S2. ^{13}C NMR spectra of a) magnolol and b) honokiol in $CDCl_3$.

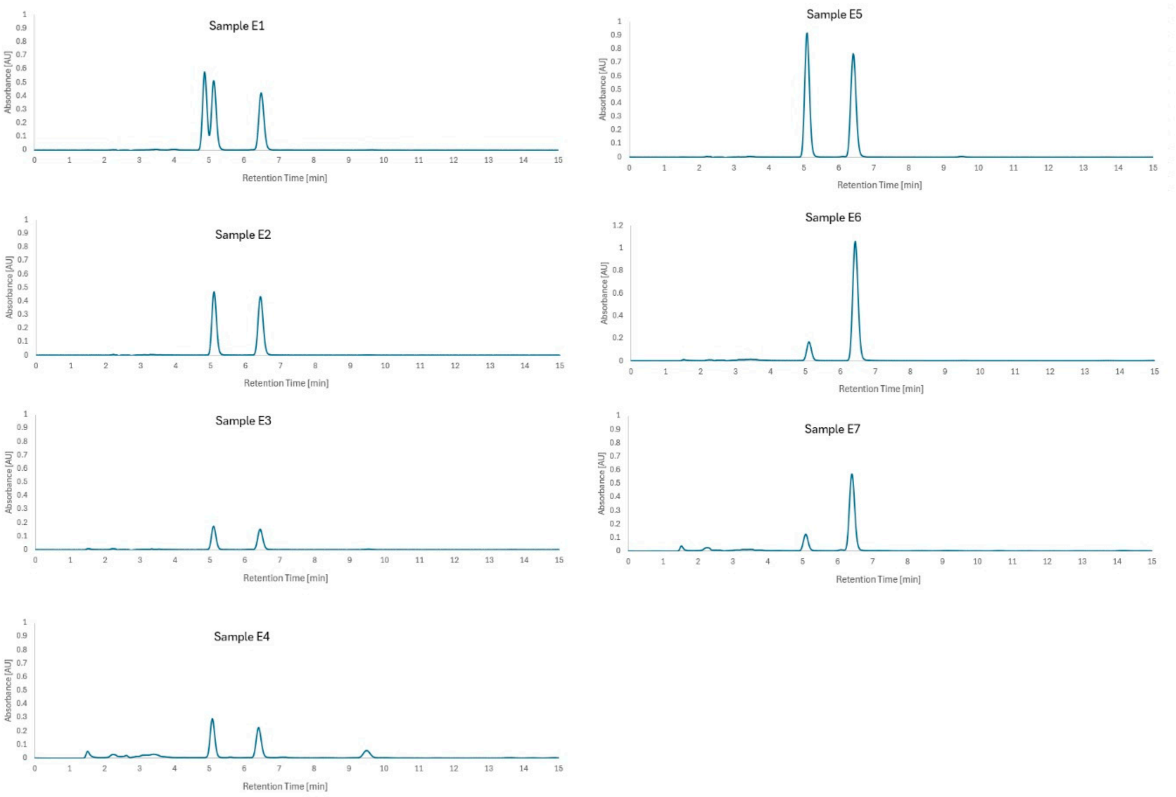

Figure S3. HPLC chromatograms of magnolia bark supplements' extracts, recorded at 290 nm.
